# The Solution Structure of the N-Terminal Domain of Human Tubulin Binding Cofactor C Reveals a Platform for Tubulin Interaction

**DOI:** 10.1371/journal.pone.0025912

**Published:** 2011-10-18

**Authors:** Mª Flor Garcia-Mayoral, Raquel Castaño, Monica L. Fanarraga, Juan Carlos Zabala, Manuel Rico, Marta Bruix

**Affiliations:** 1 Departamento de Química Física Biológica, Instituto de Química Física Rocasolano, Consejo Superior de Investigaciones Científicas (CSIC), Madrid, Spain; 2 Departamento de Biología Molecular, Instituto de Formación e Investigación Marqués de Valdecilla, Facultad de Medicina, Universidad de Cantabria, Santander, Spain; National Institute for Medical Research, Medical Research Council, United Kingdom

## Abstract

Human Tubulin Binding Cofactor C (TBCC) is a post-chaperonin involved in the folding and assembly of α- and β-tubulin monomers leading to the release of productive tubulin heterodimers ready to polymerize into microtubules. In this process it collaborates with other cofactors (TBC's A, B, D, and E) and forms a supercomplex with TBCD, β-tubulin, TBCE and α-tubulin. Here, we demonstrate that TBCC depletion results in multipolar spindles and mitotic failure. Accordingly, TBCC is found at the centrosome and is implicated in bipolar spindle formation. We also determine by NMR the structure of the N-terminal domain of TBCC. The TBCC N-terminal domain adopts a spectrin-like fold topology composed of a left-handed 3-stranded α-helix bundle. Remarkably, the 30-residue N-terminal segment of the TBCC N-terminal domain is flexible and disordered in solution. This unstructured region is involved in the interaction with tubulin. Our data lead us to propose a testable model for TBCC N-terminal domain/tubulin recognition in which the highly charged N-terminus as well as residues from the three helices and the loops interact with the acidic hypervariable regions of tubulin monomers.

## Introduction

In recent years, a great effort has been made to elucidate the complex series of events occurring during the α- and β- tubulin folding pathways that lead to the final release of αβ native heterodimers incorporated in microtubules [1], [2]. In mammals, this process is initiated by the cytosolic chaperonin CCT (also known as c-cpn or TriC) binding to the newly synthesised α- and β-tubulin polypeptides [3] assisted by the molecular chaperone protein prefoldin that, after various ATP-hydrolysis-dependent cycles, produces quasi-native tubulin intermediates. In contrast to actin and γ-tubulin that can be completely folded by the exclusive action of chaperonins, the intermediates of α- and β-tubulin need to be further processed to reach their final active conformation, a process that requires a set of five different tubulin binding cofactors (TBCA, TBCB, TBCC, TBCD, and TBCE). TBCB associates with α-tubulin folding intermediates and is then displaced by TBCE. TBCA and TBCD interact in a similar way with quasi-native β-tubulin. An additional tubulin binding cofactor, TBCC [4], is necessary to complete the process by forming a supercomplex with TBCD, β-tubulin, TBCE, and α-tubulin that, following GTP-hydrolysis-dependent cycles, releases the native αβ-tubulin heterodimers. The stimulated hydrolysis of GTP by β-tubulin acts as a switch for the release of native tubulin heterodimers from the supercomplex [5]. The discovery of this pathway has driven much of the effort to the study of the implication of these proteins in the folding/dimerization of tubulin.

Recent results have shown that tubulin binding cofactors also participate in the proteostasis of the tubulin dimer through their intrinsic ability to dissociate the tubulin heterodimer [1], [2]. This ability to dissociate the tubulin heterodimer in a controlled way is a mechanism that certain types of cells exploit to regulate key cytoskeletal processes, such as controlling their microtubule densities, or the trimming of the distal microtubule tips at the axonal growth cone terminal in macrophages and neurons respectively. TBCC is probably the least understood tubulin binding cofactor and no reports regarding its function *in vivo* have been published.

**Figure 1:**
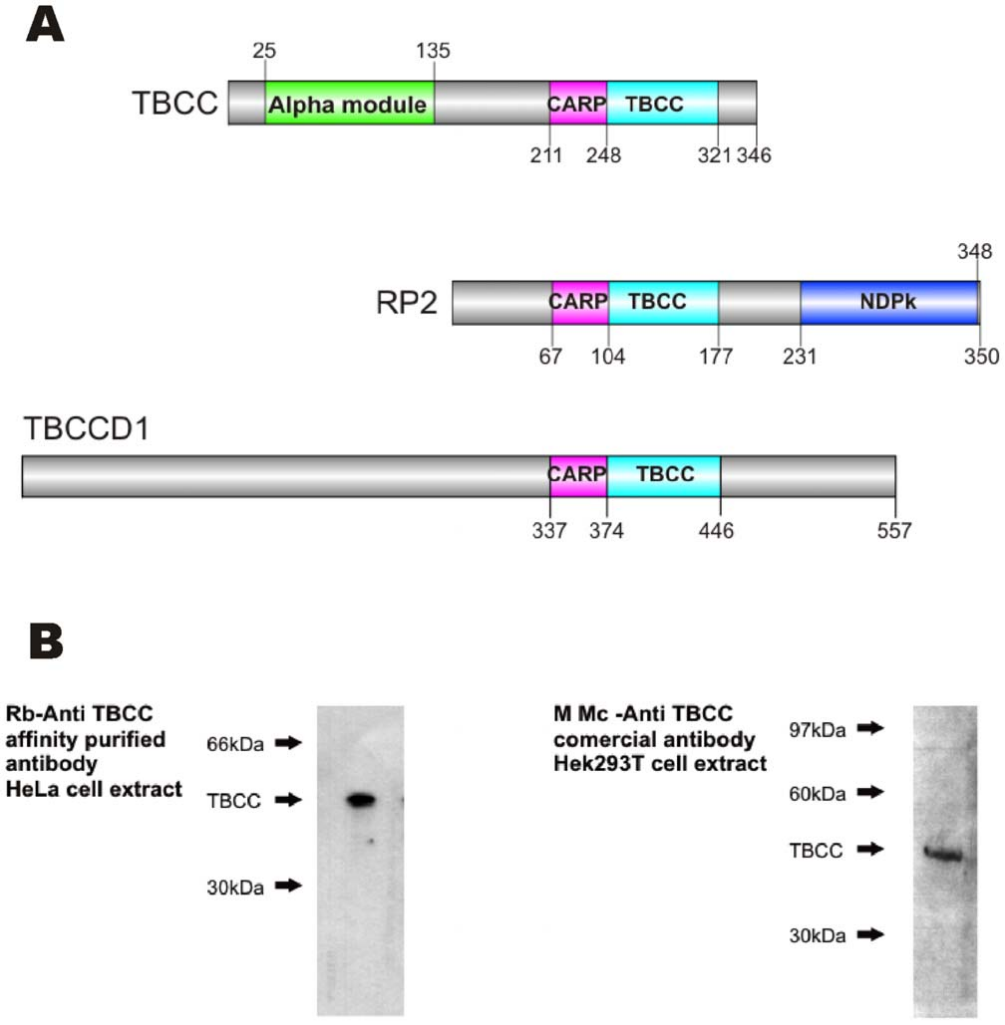
Specificity of polyclonal and monoclonal anti-TBCC antibodies. A) Human TBCC protein family. Schematic representation of the functional domains ascribed to human TBCC, RP2 and TBCCD1. The human proteins also possess a CARP domain present in CAPs (cyclase-associated proteins) [58]. TBCCD1 is related to TBCC and RP2 which functionally overlaps with TBCC [6]. The C-terminus known as the TBCC domain is shown in light blue, the CARP domain in magenta and the N-terminus domain (alpha module) in green. B) Both, the purified rabbit polyclonal anti-TBCC produced in our laboratories (left) and the commercial mouse monoclonal antibody also used in this study (right, see Methods) recognised a single band in whole cell extracts.

TBCC is organized into three different domains (N-term, CARP and C-term) (Fig. 1A). The C-terminal domain constitutes the hallmark of the TBCC protein family and its structure was recently solved by Saito, K. et al. (2007, PDB: 2YUH). This domain shares ∼29% sequence identity over half of the length of Retinitis Pigmentosa 2 protein (RP2) and both proteins stimulate the GTPase activity of native tubulin with the cooperation of TBCD. In contrast to TBCC, RP2 has no tubulin heterodimerization capacity [6]. This domain is also present in TBCCD1 (TBCC-domain containing 1), a protein that localizes at the centrosome and basal bodies of primary and motile cilia, required for centrosome and Golgi Apparatus (GA) positioning in human cells [7], [8]. The TBCC C-terminal domain has a conserved arginine (R262) also present in RP2 (R118) postulated to act as an arginine-finger in the GTP hydrolysis of tubulin in similar manner as the arginine-finger in RasGAP [9]. Like the corresponding mutation in RP patients, substitution of R262 of TBCC abolishes its GTPase activating protein (GAP) activity suggesting a role in regulation of microtubule polymerization *in vivo* [6].

Although the N-terminal domain is expected to interact with other spectrin-like domains [10], no functional roles have yet been assigned.

In this work we have demonstrated that TBCC is found at the centrosome and we have used NMR spectroscopy to determine the solution structure and the interactions with the αβ-tubulin dimer of its N-terminal domain (TBCC N-terminal domain: residues 25–135).

## Results

### TBCC is found at the centrosome

**Figure 2:**
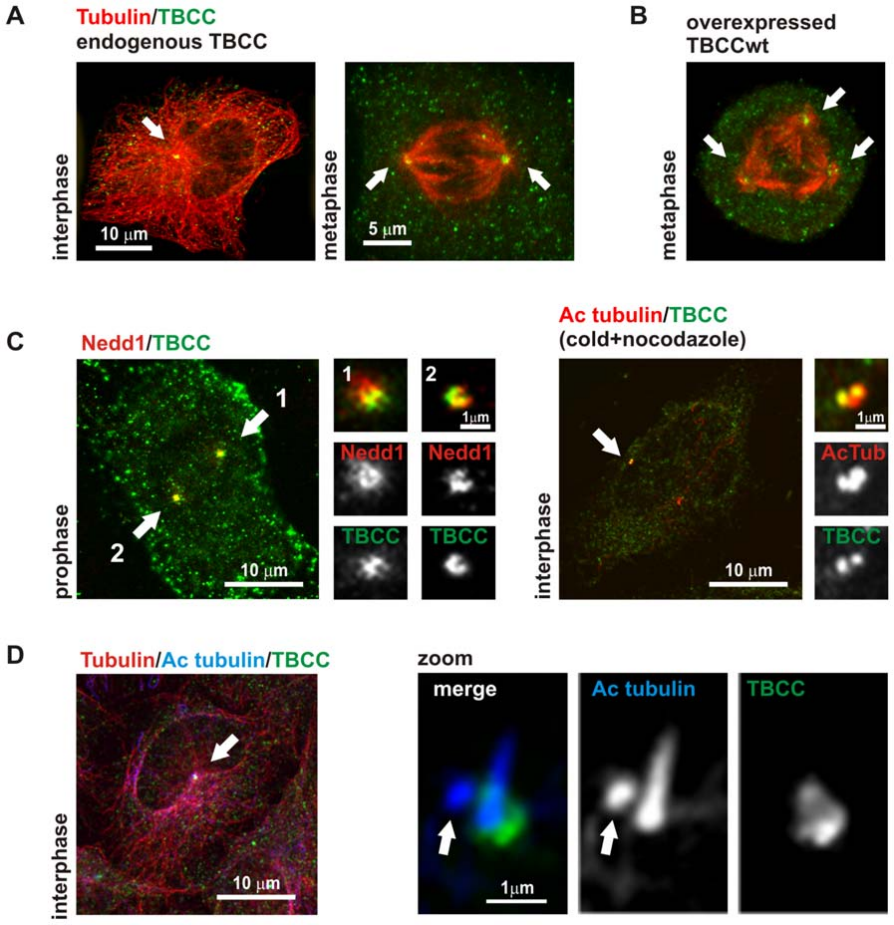
TBCC is located at the centrosome. A) Confocal microscopy image of TBCC localization on interphase (left) and mitotic (right) HeLa cells. TBCC is mostly a cytoplasmic protein but concentrates at the centrosomes of HeLa cells (arrows). B) TBCC overexpression produces an increase of TBCC immunostaining at the spindle poles (arrows) and a higher rate of mitotic aberration defects such as multipolar spindles. C) (left) HeLa cells in prophase exhibiting a clear TBCC colocalization with the protein Nedd1, a classical centrosomal marker. High resolution confocal microscopy images of both centrosomes (#1,#2) are shown. (right) Confocal-microscopic image of HeLa cells where the microtubule cytoskeleton has been destroyed by cold and nocodazole treatment. Double-immunostaining against acetylated tubulin and TBCC revealed how, under these conditions, TBCC colocalizes with the centrioles that typically exhibit acetylated tubulin. D) (left) Triple immunostained HeLa cell displaying a primary cilium (arrow). (right) High resolution confocal image of the primary cilium and daughter centriole (arrow) immunostained with anti-acetylated tubulin (blue channel) and TBCC (green channel). These images show that TBCC is mostly localised at the base of the primary cilium, around the basal body.

**Figure 3:**
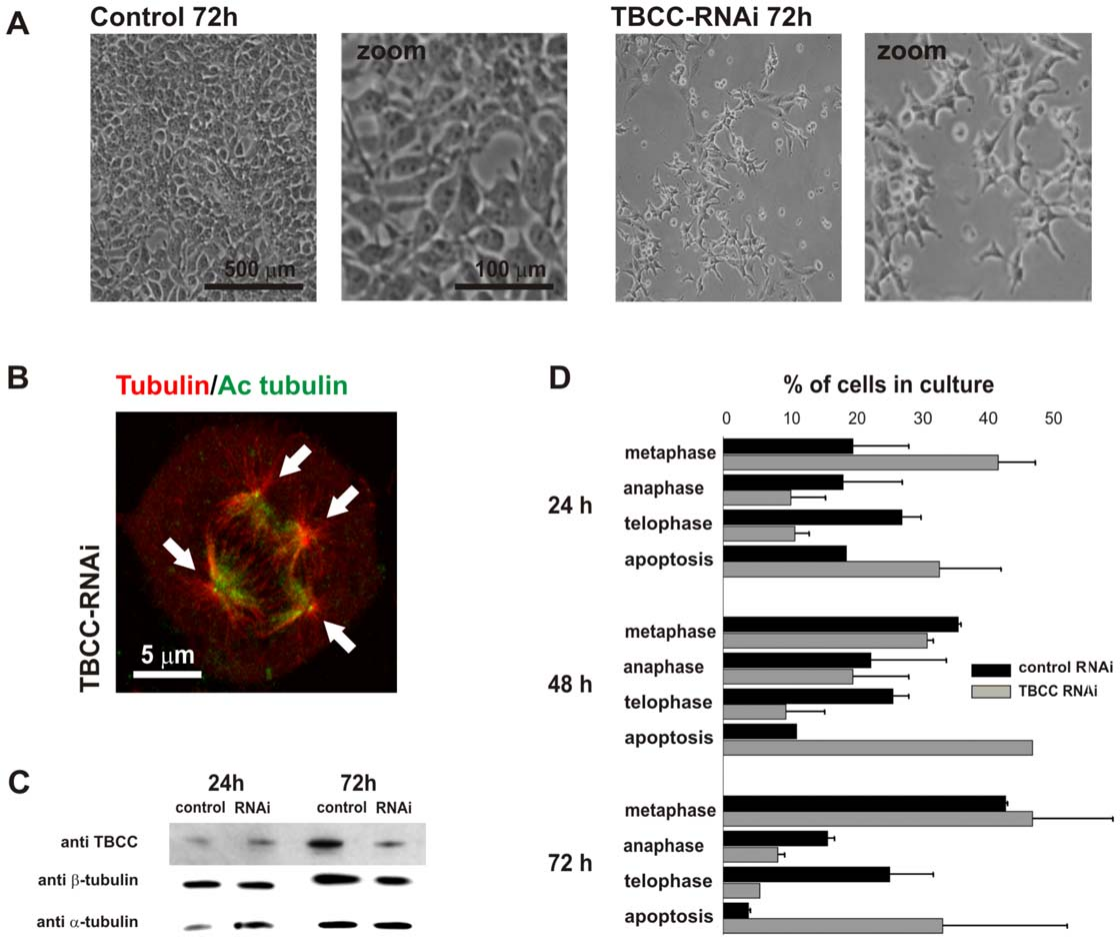
TBCC depletion leads to mitotic failure and apoptosis. A) (left) HeLa cell culture treated with control RNAi for 72 h. A cell confluence of almost 90% is achieved. (right) Identical culture treated with TBCC RNAi for 72 h. TBCC gene interference produces a rise in cell death leading to a conspicuous cell depletion in the culture. B) Confocal-microscopy projection image of 72 h RNAi treated HeLa cell where a multipolar mitotic spindle is shown (spindle poles indicated by arrows). C) Western blot confirmation of TBCC silencing on whole cell lysates (50 µg/lane total protein). TBCC expression was compared to total α- and β-tubulins. TBCC depletion did not noticeably affect tubulin levels at this post-transfection time point. D) Distribution of the different mitotic cell stages observed in TBCC RNAi treated cultures and controls at different time points after TBCC RNAi treatment. These data show that TBCC RNAi treatment blocks cells mostly at metaphase, leading to a high rate of apoptotic cells. Data represent mean values and bars standard errors.

**Figure 4:**
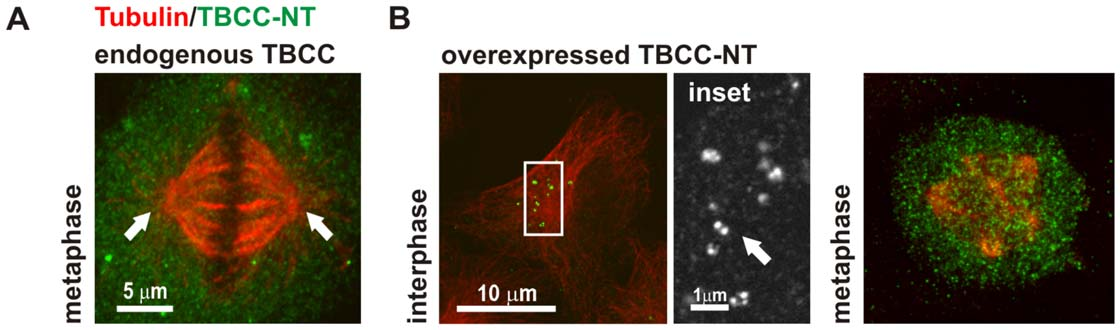
The TBCC N-terminal domain is embedded at the centrosome. A) Mitotic HeLa cell doubly-immunostained with the anti-TBCC antibody purified against the TBCC N-terminal domain, and tubulin. TBCC (arrows) is not detected at the centrosome by the antibody purified against the N-terminus of TBCC (immunoglobulins recognizing the C-terminus are removed). This result suggests that the TBCC N-terminal domain centrosomal epitopes are masked in the centrosome. B) (left) High resolution confocal images of HeLa cells transfected with the TBCC N-terminal domain. Overexpression of the TBCC N-terminal domain produces accumulates of this protein at the perinuclear-centrosomal region (inset, arrow). (right) Confocal microscopy projection image of a mitotic HeLa cell transfected with the TBCC N-terminal domain and doubly immunostained against tubulin and TBCC. TBCC N-terminal domain overexpression produces mitotic aberration defects such as multipolar spindles, similar to those observed for the full-length construct.

To study TBCC function, we investigated the subcellular distribution of the endogenous protein in HeLa cells with a novel affinity antiserum purified against the human recombinant protein. The primary antibody recognizing human TBCC used was affinity purified as previously described [11] against both, the full length protein (Fig. 1, 2 and 3) or the TBCC N-terminal domain (Fig. 4) to select TBCC N-terminal recognizing immunoglobulins from the antiserum. A commercial anti-TBCC monoclonal antibody (Abnova Corporation) was used to validate the TBCC centrosomal immunostaining pattern. These antibodies recognised a unique protein band corresponding to TBCC in western blots (Fig. 1B). Doubly immunostained cells revealed a dot-like cytoplasmic labelling accompanied by a prominent and irregular centrosomal spot of TBCC (Fig. 2A). A centrosomal immunostaining pattern was also observable in metaphase cells where both spindle poles displayed TBCC accumulation (Fig. 2A right, arrows).

We next overexpressed TBCC in order to investigate TBCC subcellular localization. We observed accumulates of this cofactor at spindle pole bodies and occasional multipolar spindles (Fig. 2B). These results match those observed by Hage-Sleiman et al. [12] in MCF7 cells (human mammary adenocarcinoma), where a G2-M phase blockage in TBCC overexpressing cells has been reported.

Based on these findings, we hypothesize that TBCC is localised at the centrosome. We compared TBCC colocalization with classical centrosomal markers, such as γ-tubulin (not shown) or Nedd1, and as Fig. 2C (left) shows, TBCC produced an overlapping immunostaining pattern thus supporting our hypothesis. But since centrosomal proteins are typically recognized by colocalization with centrosomal/centriolar markers after microtubular destruction by cold and nocodazole, we destroyed the microtubule cytoskeleton to corroborate the above hypothesis. Fig. 2C (right) shows that TBCC was still detectable at the centrosome of cold and nocodazole treated HeLa cells, partially colocalizing with the centrioles labelled with an anti-acetylated tubulin antibody. Moreover, HeLa cells displaying a primary cilium (Fig. 2D) exhibited TBCC at the base of the basal body (mother centriole) rather than the daughter centriole (arrow).

Subsequently, we silenced TBCC gene expression with a pool of four synthetic RNAs recognizing different segments of the TBCC mRNA and specifically designed to knockdown the human TBCC gene with no off-target effect (see Methods). As Fig. 3A shows, a noticeable reduction in cell numbers was clearly observed after 72 h treatment with TBCC RNAi. TBCC gene downregulation produced a broad range of mitotic spindle defects and mitotic failure (Fig. 3B, ) typically reported for most centrosomal proteins [13]. On the other hand, the severe depletion observed for this protein in whole HeLa cell extracts was however not accompanied by a marked reduction in α- and β-tubulin levels (Fig. 3C). A quantitative and morphological study of these cultures revealed a high proportion of cells blocked at mitosis as soon as 24 h after RNAi treatment (Fig. 3D), a result which was further supported by a reduced number of cells undergoing anaphase and telophase, and a higher apoptotic rate compared to controls. Moreover, less than 20% of the mitotic cells in TBCC RNAi treated cultures displayed standard bipolar metaphases, while almost 30% displayed evident aberrant mitotic figures, mostly multipolar spindles. Longer RNAi incubation times (48 and 72 h) as shown above, produced a massive rise in cell death. These data support the hypothesis that TBCC is a key protein in centrosomal function at mitosis.

### The TBCC N-terminal domain is masked at the centrosome

As part of the original study, we also affinity purified the same rabbit polyclonal antiserum against the N-terminal domain of TBCC. Unexpectedly, the same antisera, when purified against the TBCC N-terminal domain, produced a similar cytoplasmic immunostaining pattern but did not label the centrosome (Fig. 4A, arrows). These differences suggest that the TBCC N-terminal domain is masked at the centrosome.

In the view of the above results, we decided to study a TBCC truncation mutant containing the N-terminal domain overexpressed in HeLa cells. In contrast to the cytoplasmic pattern observed for the full-length polypeptide, the TBCC N-terminal domain produced a dot-like pattern, distributed at the perinuclear-centrosomal region (Fig. 4B left). As observed for the full-length construct, TBCC N-terminal domain overexpression was also associated with a number of metaphase aberrations (Fig. 4B right). These results confirm a role for TBCC at the centrosome and support the hypothesis that the TBCC N-terminal domain is masked within this organelle. These data led us to study in more detail the TBCC N-terminal domain.

### Structure of the TBCC N-terminal domain

**Figure 5:**
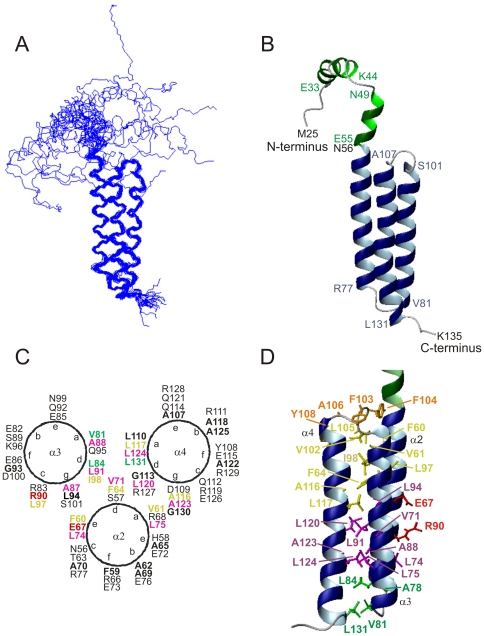
Solution structure of the TBCC N-terminal domain. A) Superposition of the 20 lowest-energy conformers. B) Ribbon display of a representative conformer of the family showing the limits of the helical segments and one of the possible orientations of the N-terminal tail (in green) with respect to the protein core. Two regions with helical propensity (33–44) and (49–55) are labelled. C) Hydrophobic contacts in the interior of the bundle. Different colours are used along the helix axis for the upper N-terminal side (yellow), the lower C-terminal side (magenta), the bottom part including loop 2 (green), the top part comprising loop 3 next to the disordered N-terminal region (orange). The salt bridge between E67 and R90 is highlighted in red. D) Distribution of aliphatic/aromatic and charged/polar residues along the helices. The hydrophobic side-chains are concentrated at the helical interfaces favouring the molecular packing, and most polar and charged residues are in contact with the solvent. Colour code as in C).

Fig. 5A shows the superposition of the 20 conformers of the TBCC N-terminal domain determined by NMR. The structure is a left-handed 3-stranded α-helix bundle composed of 3 antiparallel and almost coaxial α-helices: α2, N56-R77; α3, V81-S101; α4, A107-L131 connected by short linkers: loop 2, A78-S80; loop 3, V102-A106. The N-terminal portion (residues 25–55) of this domain has not a defined orientation relative to the protein core and shows regions with partial helix formation (Fig. 5B). In particular, residues E33-K44 and N49-E55 adopt helical conformations with populations of ∼60 and ∼38%, respectively as estimated on the basis of their conformational shifts [14]. No NOEs connect these nascent helices to the rest of the protein. The entire N-terminal region is structurally disordered relative to the domain and samples all the available conformational space. The structured part of the protein (residues 56–131), is well-defined with low pairwise RMSD values (Table 1). Average interhelical angles of 170° between helix α2 and α3, 6° between helix α2 and α4, and 173° between helix α3 and α4 are obtained for the ensemble. The compact helix bundle confers the molecule a rod-like shape with a volume of 11000 (∼17×17×38) Å^3^ and a global accessible surface area of 6400 Å^2^ [15]. Helical wheel projections (Fig. 5C) show that the sequences of the three helices conforming the TBCC's bundle fulfil the characteristic heptad pattern of left-handed coiled coils [16].

**Table 1:** Structural Statistics of the 20 Best NMR Structures of the TBCC N-terminal domain.

**NOE Distance and Dihedral Constraints**	
No. of short-range distances (|i-j|≤1)	990
No. of medium-range distances (1<|i-j|<5)	412
No. of long-range distances (|i-j|≥5)	290
No. of angular restraints (ϕ, ψ)	95
No. of total restraints	1692
**Structure Calculation**	
Average CYANA target function value	0.49
Maximum distance violation (Å)	0.25
Maximum dihedral angle violation (°)	2.87
Average AMBER energy (kcal/mol)	−7000
**RMSD (Å)**	
Bond lengths from ideal geometry	0.0108±0.0006
Bond angles from ideal geometry	1.99±0.02
Pairwise backbone (26–135)	7.63±2.75
Pairwise heavy atom (26–135)	8.79±2.87
Pairwise backbone (56–131)	0.65±0.13
Pairwise heavy atom (56–131)	1.53±0.14
**Ramachandran Plot Analysis (%)**	
Most favored regions	94.7
Additional allowed regions	5.3
Generously allowed regions	0
Disallowed regions	0

The side chains of a significant number of hydrophobic residues are deeply buried in the protein core, pointing to the interior of the helix bundle (Fig. 5C, 5D). Among them, F60, F64, L75, A78, L84, A87, L91, L94, I98, L105, G113, A116, L117, L120, L124 possess ASA values below 5%. These residues participate in hydrophobic interactions that contribute to stabilizing the helical bundle by forming an extended hydrophobic platform along the helix axis. These interactions include the aromatic-aromatic contacts between F60 and F64 in an edge-to-face fashion, and many aromatic-aliphatic and aliphatic-aliphatic contributions. For example, close contacts (<5 Å) in the upper part of the bundle involve the aromatic rings of F60 and F64 with the aliphatic chains of V61, L94, L97, I98, V102, L105, A116, and L117 (Fig. 5C, 5D, yellow). In the lower part of the bundle, interactions involve V71, L74, L75, A87, A88, L91, L94, L120, A123, L124 also from the three helices (Fig. 5C, 5D, magenta). At the bottom A78, V81, L84, L131 belong to loop 2 and the N- and C-termini of helices α3 and α4, respectively (Fig. 5C, 5D, green). Close to the disordered part, the aromatic rings of F103 and F104 interact with the methyl groups of V102, L105 and A106, all located in loop 3 (Fig. 5C, 5D, orange). Y108 is also close to A106 (∼4.6 Å). In contrast to hydrophobic interactions, electrostatic interactions are much less abundant. A salt bridge connecting the side-chains of E67 and R90, that links helices α2 and α3 (Fig. 5C, 5D, red), is present. The side chains of residue pairs E76-R127, E79-R83, E82-R83, E126-R127, and E126-R129 are relatively close to each other and may form favourable charge-charge interactions.

**Figure 6:**
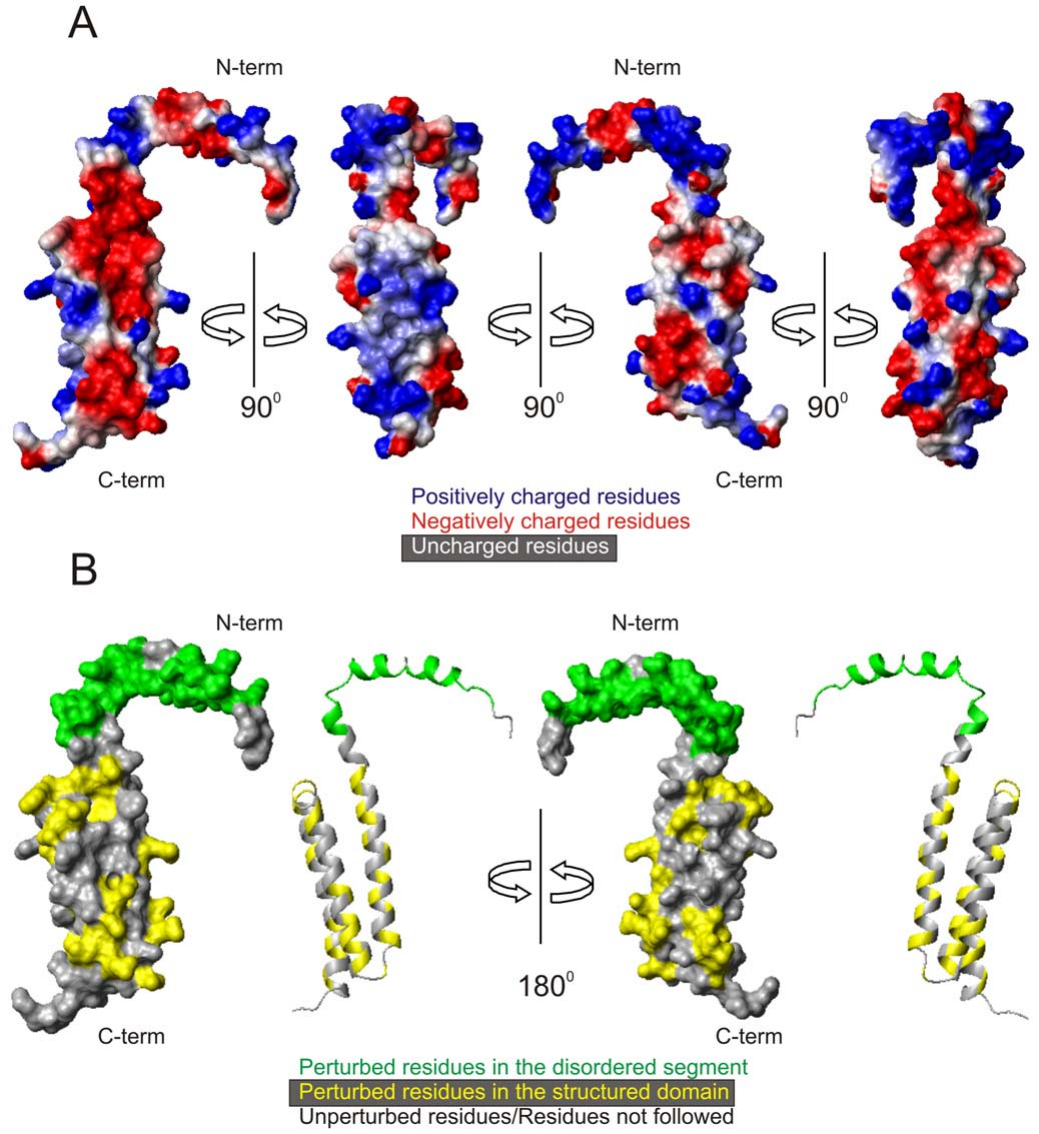
Surface properties of the TBCC N-terminal domain. A) The electrostatic surface is represented for four views corresponding to 90° rotations. The distribution of the negatively charged (red), positively charged (blue), and nonpolar residues (white) defines a highly charged surface, with two 90°-rotated faces concentrating mainly negative and positive charges (left), and the other two with more random charge distribution (right). B) Two 180° rotated views of the mapped chemical shift perturbation data. Residues affected by the interaction with αβ-tubulin dimer are coloured in yellow in the helices and in green for the N-terminal disordered segment.

The surface of the TBCC N-terminal domain is highly charged (Fig. 6A). Interestingly, two contiguous regions differing in 90° rotation concentrate longitudinally charges of opposite signs while on the two remaining faces there is a more random distribution. Such a distribution would favour protein-protein interactions with partners having the appropriate charge complementarities. Also, remarkably, the 30-residue N-terminal region is very rich in positive charges, except for a central patch of negatively charged residues (E33, E35, E39, and E41). The 30-residue N-terminal region concentrates 80% of charged and polar residues with ASAs≥30% and these features are likely to be important for the interaction with tubulin as discussed below.

### Dynamics of the TBCC N-terminal domain

**Figure 7:**
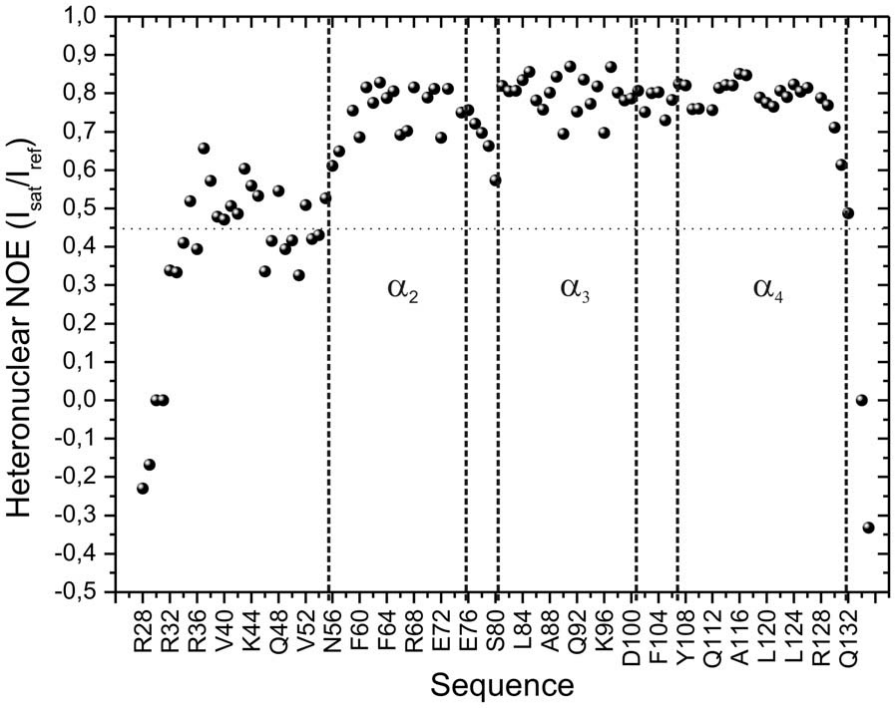
TBCC N-terminal domain heteronuclear NOEs for local backbone flexibility. Residues in the 30-residue N-terminal segment have lower than average NOE values, indicative of high backbone mobility in the ns-ps time-scale. Some flexibility is also found at the C-terminus of the domain and residues at the interhelical connecting loops. The dynamics of the helices α2, α3, α4 is more restricted.

We have measured the heteronuclear NOEs to get information on the local backbone flexibility of the TBCC N-terminal domain in the ns-ps time-scale (Fig. 7). The dynamics of helices α2, α3, and α4 is quite restricted although several residues located at both termini of helix α2 (N56, S57, F60, R77) as well as those in loop 2 (A78-S80), L105 in loop 3, residues G130 and L131 at the end of helix α4, and the C-terminus are more flexible. Interestingly, the 30-residue N-terminal region is highly dynamic; all residues display lower than average heteronuclear NOEs. These data corroborate the disordered nature of the N-terminal region, and shows that its high flexibility is responsible for the absence of long range NOEs.

### Interaction of the TBCC N-terminal domain with tubulin

**Figure 8:**
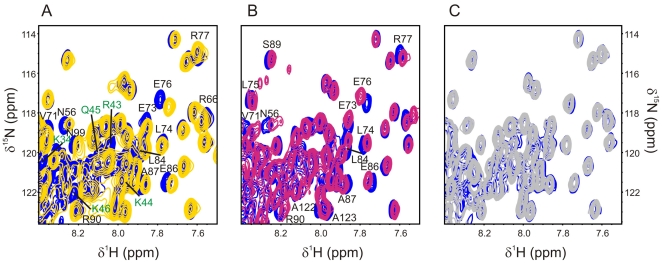
TBCC N-terminal interaction assays with αβ-tubulin dimer and C-terminal β-tubulin peptides. Superposition of ^15^N-HSQC spectra of the TBCC N-terminal domain free (blue) and in the presence of αβ-tubulin heterodimer (yellow). Selected perturbed residues are labelled, with green labels corresponding to amino acids in the N-terminal disordered region. **B**) Superposition of ^15^N-HSQC spectra of the TBCC N-terminal domain free (blue) and in the presence of an excess of the 16-residue C-terminal β-tubulin peptide EMYEDDEEESESQGPK (magenta). Selected perturbed residues are labelled. **C**) Superposition of ^15^N-HSQC spectra of the TBCC N-terminal domain free (blue) and in the presence of excess of the 20-residue C-terminal β-tubulin peptide ESNMNDLVSEYQQYQDATAD (grey). No significant perturbations are observed.

**Figure 9:**
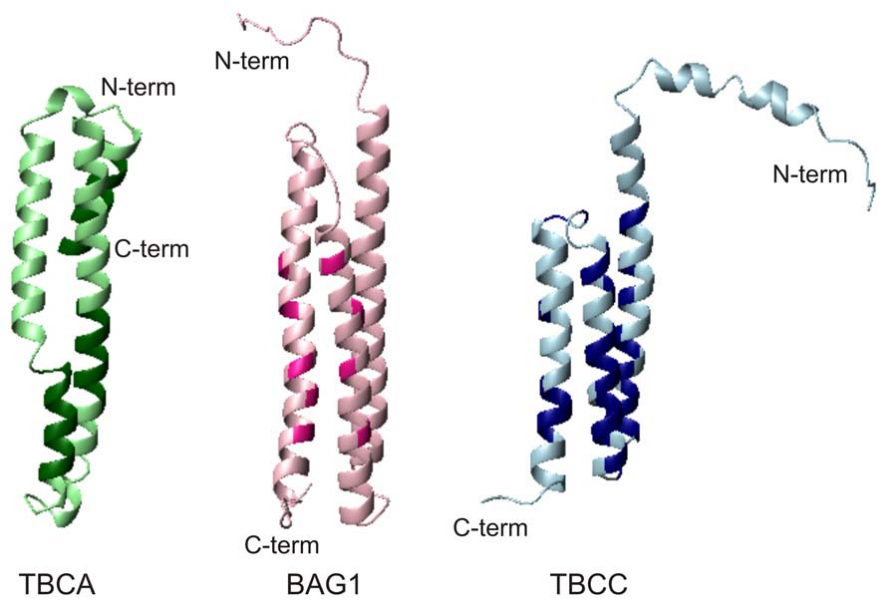
Comparison of the interacting face of TBCA, BAG1 and TBCC N-terminal domain. Ribbon displays of the similarly oriented spectrin-like domains of TBCA (left), BAG1 (middle), and TBCC (right) with the residues involved in the interaction with β-tubulin, the ATPase domain of Hsc70, and the 16-residue C-terminal β-tubulin peptide EMYEDDEEESESQGPK (435–450), respectively, shown in strong colours.

We tested whether the TBCC N-terminal domain is able to interact directly with αβ-tubulin heterodimer and with two peptides of 16 (residues 435–450, 9 residues charged, 8 negatively and 1 positively charged) and 20 (residues 412–431, includes helix H12, 5 residues negatively charged) residues derived from the C-terminus of the β6-tubulin subunit (class III, [17]). Region 412–431 is highly conserved in tubulins and the last 10–15 residues of their C-terminus represent the most variable region although it is always negatively charged and contains several Glu residues. Secondary structure predictions and circular dichroism experiments suggested that while the region 412–431 forms an α-helix, the last 10–15 C-terminal residues lacks ordered structure [18] independently of the isotype. This last region of β-tubulin is known to interact with many microtubule associated proteins (MAPs) and the electrostatic contacts with the stretches of negatively charged residues have been reported to play a crucial role in the interaction [18], [19], [20]. At this regard, the 16-aminoacid peptide was chosen to specifically include the acidic region. We monitored the changes induced in the ^15^N-HSQC spectra of the labelled TBCC N-terminal domain in the presence and absence of unlabelled αβ-tubulin and β-tubulin peptides (Fig. 8). Severe spectral broadening most likely accompanied by the consequent loss of signals would be expected upon the formation of the large TBCC-tubulin complex. For this reason we have explored the interaction with low TBCC/tubulin ratios, conditions that can be followed by NMR. Important changes in the position and the intensity of many peaks are observed upon αβ-tubulin titration that map to a large portion of the surface of the TBCC N-terminal domain (Fig. 8A and Fig. 6B). Interestingly, at the lowest amounts of tubulin, there was a set of residues whose signals considerably broadened, including N56, F104, A107, and S57, which almost disappear. All observed changes map to an interaction surface that includes the disordered and flexible 30-residue N-terminal segment, as well as residues from the three helices, and the loops (Fig. 6B). Such a large contact surface is probably indicative of significant conformational changes occurring upon binding. Many of the residues affected are charged and polar, particularly those in the 30-residue N-terminal segment. Examples of these residues in helices α2–α4 and loops are: N56, S57, E73, E76, R77, E79, E82, E85, E86, R90, N99, D100, Q114, and E126. Also, important hydrophobic groups from V61, F64, A69, V71, L84, A87, L91, F103, F104, A106, A107, A122, A123, and A125, participate in binding. Most of these residues are also perturbed by the interaction with the 16-residue peptide corresponding to the tubulin sequence 435–450 (Fig. 8B and Fig. 9) but no spectral changes occur with the 20-residue peptide corresponding to the tubulin sequence 412–431 (Fig. 8C), suggesting that the sequence region covered by the latter peptide does not participate directly in binding.

## Discussion

To date not much literature is available on the biological processes involving TBCC. It is known to be involved in the last step of the pathway leading to correctly folded αβ-tubulin, however the molecular details of the mechanistic aspects remain elusive. This protein is necessary for life in higher eukaryotes [21], [22], [23], [24], [25] and has been implicated in cancer cell proliferation control [12], but so far no reports regarding its function *in vivo* have been published.

### Comparison of TBCC with other tubulin cofactor structures

From the set of five cofactors assisting tubulin folding, structural data are available for only two of them, cofactors A and B of different species. Both form stable complexes with β-tubulin and α-tubulin folding intermediates, respectively, in the presence of GTP [26] following their release from the αβ-tubulin-CCT binary complex [27]. Human cofactor A, the first cofactor discovered to participate in the process [28], [29], is the one whose structure most closely resembles the N-terminal domain of TBCC although it has not sequence similarity and lacks disordered regions (Fig. 9). Its X-ray structure revealed a helical bundle of three antiparallel helices [30] similar to that of the TBCC N-terminal domain, although their lengths are quite different. The crystal structure of the TBCA yeast homolog in *S. cerevisiae*, Rbl2p, shares a similar overall fold but crystallizes as an inverted dimer with the two long helices associated into a four-helix bundle [31]. On the other hand, the crystal structure of *A. thaliana* TBCA has recently been reported [32]. It forms a monomeric three-helix bundle where the α-helical residues are important for β-tubulin affinity as was previously shown for the human ortholog [30]. A search for structural homologs of TBCA yielded the cytoskeletal proteins α-spectrin and α-actinin. The length of the rod units is identical in TBCA, BAG1 domain (Fig. 9) and spectrin/actinin repeats [30]. It is noteworthy that recently, PRC1, a nonmotor microtubule binding protein belonging to the MAP65 protein family, has been found to establish crosslinks in dynamic cytoskeletal networks. These proteins have an N-terminal coiled-coil domain, a C-terminal regulatory domain and a central region that mediates microtubule binding. This last domain also resembles spectrin repeats structurally [33]. While MAP65 proteins bind to microtubules, TBCA binds to the β-tubulin subunit.

### Biological role of the flexible disordered N-terminal segment of the TBCC N-terminal domain

Interestingly, some of the cofactor structures have disordered regions for which roles in intermolecular interactions have been proposed. The crystal structure of the CAP-Gly domain of TBCB in *C. elegans*, F53F4.3, shows a completely different fold with three antiparallel β-sheets [34]. However, despite this different topology with respect to the TBCC N-terminal domain, it also has a flexible α-helix at the N-terminus preceded by 17 disordered residues which were proposed to participate in intermolecular interactions [34]. The TBCC homolog RP2 has a chiefly disordered 33 residue segment at its N-terminus that was also proposed to participate in interactions [35].

Using NMR chemical shift perturbation mapping, we have shown that the region of the TBCC N-terminal domain involved in tubulin binding includes the flexible 30-residue N-terminal segment, which remarkably concentrates the largest number of charged and polar residues. The calculated pI value for this region, 9.7, reflects a markedly cationic character suggestive of a possible involvement in electrostatic interactions with the C-terminal anionic domains of tubulin. A pair of adjacent Arg and Lys residues have been shown to be essential for the ability of the microtubule destabilizing domain of centrosomal protein CPAP to bind to microtubules [36]. Also, site directed mutagenesis corroborated the importance of positively charged residues in the binding of CLIP170 [37]. At this respect, it is of note that the stretch 42–47 of the TBCC N-terminal domain flexible segment (RRKQKR) has a high local positive charge density and would be a good candidate for target interactions. Moreover, we have provided evidence that the 30-residue N-terminal segment of the TBCC N-terminal domain is highly mobile and disordered in the free protein according to NMR relaxation measurements. High degrees of disorder and unfolded segments are a common feature of many centrosomal proteins [38] suggesting that this might be an intrinsic requirement for biological function. Nowadays there is considerable evidence that microtubule associated proteins are unstructured in solution [39], [40], [41] and become ordered upon binding to protofilament surface [18]. Although the 30-residue N-terminal region of the TBCC N-terminal domain is globally disordered with respect to the protein core, non-negligible helical tendencies exist particularly in the E33-K44 and N49-E55 segments. This flexible segment might fold into a defined tertiary structure with increased helix content upon tubulin binding in a similar way as proposed for the predominantly helical 23-residue region of the mainly unstructured PN2-3 fragment of centrosomal protein CPAP [38].

### Interaction of the TBCC N-terminal domain with tubulin

It is well known that NMR spectroscopy provides a fast method for analysing weak protein–protein interactions [42] and is therefore highly convenient for the study of transient protein complexes that are difficult to detect by other methods. Specifically, monitoring chemical shift perturbation is the most widely used NMR method to map protein interfaces [43]. The tubulin binding region mapped in this study for the TBCC N-terminal domain (Fig. 6B and Fig. 9) partially agrees with that suggested for TBCA or BAG1 proteins with β-tubulin and the ATPase domain of Hsc70, respectively. In the present study, the interaction has been corroborated with synthetic peptides covering relevant regions of the β-tubulin sequence (412–431 and 435–450) and with the tubulin heterodimer. In our study, the sequence of the peptide 435–450 was derived from the human β6 tubulin isotype (Class III) found in humans by Sullivan and Cleveland [17]. Although there might be some small differences (one residue) with respect to the sequences in the databases, the classical stretch of negatively charged residues is present in all isotypes. Also, TBCC should be promiscuous regarding β-tubulin isotype binding because there is just one TBCC gene in the genome and it contains a single exon. We do not know the actual TBCC partner at the centrosome but it is noticeable that TBCC binds to different tubulin isotypes in the supercomplex formed with TBCD and TBCE during dimer formation in the postchaperonin tubulin pathway [2]. The fact that the interacting region with tubulin mapped along the helical bundle in this study (Fig. 6B) is the same as that detected with the isolated peptide (Fig. 9), strongly suggests that the interactions are physiologically relevant.

The interaction site for BAG1, also based on NMR titration data with synthetic peptides that mimic some helices of the ATPase Hsc70 subdomains, was assigned predominantly to the central regions of helices α2 and α3 (Fig. 9), on the same face of its conserved domain [44]. For TBCA, although interacting regions were mapped along the three helices using binding assays of β-tubulin with a cellulose-bound TBCA peptide library, the role of residues in helix α2 was found the more relevant [30]. Although TBCA, BAG1 and the TBCC N-terminal domain share a similar fold topology, their helix lengths and sequences greatly differ, making it difficult the identification of structurally equivalent residues for comparisons. However, our NMR data reveal a large number of residues at the contact surface along the main axis of the helical bundle suggesting that the recognition of tubulin by the TBCC N-terminal domain probably has an orientation similar to that of TBCA and BAG1. This similarity is shown in Fig. 9, where the proposed interacting surfaces for the three proteins are displayed in a similar orientation. Despite the different sequences involved, a large variety of amino acid types seems to be perturbed upon binding suggesting a complex network of intermolecular contacts responsible for the interaction with tubulin.

A significant difference is provided by the flexible N-terminal region of TBCC also participating in binding, which is neither present in TBCA nor in BAG1. A reasonable explanation for the concomitant changes observed in the flexible N-terminal segment, and nearby residues, would be a structural reorganization in which this region adopts an ordered and defined orientation within the helix bundle mediating molecular recognition. This process is most likely driven by electrostatic interactions. Interestingly, a theoretical coiled-coil structure, which includes residues in the natively disordered N-terminal end (from P26 to E55), is predicted by the program COILS [45]. In fact, the region comprising residues Q30-N56 has a high probability to adopt a helix coiled-coil structure just preceding the experimentally determined helix α2. In principle, two kinds of non-mutually exclusive elements of interaction have been postulated in disordered protein segments: molecular recognition features and preformed elements. Molecular recognition features are short regions that undergo a disorder to order transition that is induced by binding to their partners [46]. On the other hand, preformed elements are regions with some percentage of secondary structure population that are present in the free unstructured form and usually represent the first interacting elements, that grow and become more stable upon interacting with their partners [47]. In our case, the Q30-N56 region of the N-terminal domain of TBCC has the characteristics of a preformed structure, an helix, that is present although not 100% populated when isolated and that would adopt a more ordered coiled-coil structure upon binding. In this regard more work will be necessary to test this hypothesis.

In summary, we show that TBCC is a protein implicated in centrosomal stability particularly at mitosis. TBCC expression changes in human cells produce several mitotic spindle defects leading to mitotic failure and apoptosis. These results demonstrate that TBCC is a crucial protein in the control of the eukaryotic cell cycle, and support the hypothesis that this tubulin binding cofactor could be implicated in genomic instability and cancer. Our data show how TBCC interacts with components of the centrosome by its N-terminal domain, which is masked within this organelle. We have also shown that the structure of the TBCC N-terminal domain solved by NMR adopts a spectrin-like fold and with a flexible and disordered N-terminal segment. This segment is highly charged and participates in tubulin interaction. The tubulin binding region of the structured coiled coil region resembles those proposed for TBCA and BAG1 proteins.

## Materials and Methods

### Expression and purification of TBCC N-terminal domain

Human TBCC cDNA (accession number U61234) was obtained from Dr N. Cowan (New York University, Medical Center, New York, USA). The TBCC N-terminal domain was generated by PCR (codons 25–135, corresponding to the sequence showing high homology between species) and cloned into pET3a vector (Invitrogen). The TBCC N-terminal domain was expressed in the *E. coli* BL21(DE3)/pLysS strain (Life Technologies, SA, Spain) using the T7 expression system [48] and purified from 15 L culture (in 4×500 mL batches). Upon reaching optical cell densities of ≈0.7 at 600 nm, the cells were centrifuged at 3,000× g in a JA-25.50 rotor for 15 min and pelleted. The cells were then washed with phosphate buffer and pelleted again at room temperature. The cell pellet was resuspended in ¼ volume of minimal medium containing ^15^NH_4_Cl alone or with ^13^C-glucose and was incubated to allow the recovery of growth. Protein expression was induced after 1 h by addition of isopropylthio-β-D-galactoside to a concentration of 1 mM. After a 3 h incubation period the cells were harvested. Cells were pelleted by centrifugation, washed, and stored frozen at −70°C. Pellets were resuspended in 10–20 mL of 50 mM Tris pH 8.0 with protease inhibitors. Cells were then ruptured by sonication (3 bursts of 10 seconds). After centrifugation at 25,000× g in a JA-25.50 rotor for 30 min at 4°C, the supernatant was loaded onto a HiTrap Q (5 mL, GE Healthcare) equilibrated with Tris-HCl pH 8 containing 10 mM KCl. The flow-through was passed through the same column for a second time, and then applied to a high-resolution Mono-S column (5/50 GL). The TBCC N-terminal domain was eluted with a linear gradient of 10–500 mM KCl in Tris-HCl pH 8. Fractions containing the TBCC N-terminal domain were pooled, diluted 10 fold with 20 mM phosphate buffer pH 6 containing 1 mM TCEP and concentrated by ultrafiltration with Amicon Ultra 10 filters. Protein purity was determined by SDS-PAGE.

### Cell biology procedures

Passage 10 human cervical carcinoma HeLa cells cultures (obtained from EMBO laboratories stocks, Heidelberg, Germany) were fixed in chilled (−20°C) methanol or 4% paraformaldehyde, and further permeabilized in phosphate-buffered saline (PBS)–0.1% Triton X-100. For centrosomal immunostaining, microtubules were depolymerized with 2 µM nocodazole and 4°C treatments for 30 min. Anti-α-tubulin (B512) and anti-acetylated tubulin antibodies were both from Sigma (Aldrich). Human TBCC N-terminal domain was generated by PCR and inserted into the pcDNA3 vector (Invitrogene, Life Technologies). RNA interference was performed with a pool of four siRNA fragments targeting human TBCC (ONTARGETplus SMARTpool, DHARMACON, CO, USA). These siRNAs have been designed and tested to have no off-target effects. TBCC silencing was confirmed 24, 48, and 72 h after RNAi treatment by western blotting on quantified total cell extracts compared to and non target RNAi control. Morphological cell quantification (Fig. 3) was performed on live cultures to prevent cell loss during washes. Counts were performed for three different culture plates of three different experiments and controls. Statistical analysis of data and graphing were performed using the SigmaPlot 8.0 software (Systat Software, Richmond, CA). Confocal-microscopic images were obtained using a Zeiss LSM-510 confocal microscope with a 63×1, 40 lens.

### NMR, sample preparation and experiments

For NMR experiments, the ^13^C, ^15^N-labelled TBCC N-terminal domain sample was prepared in 90∶10 H_2_O∶D_2_O and D_2_O solutions of KH_2_PO_4_/K_2_HPO_4_ buffer 20 mM, 1 mM TCEP, 20 mM KCl, 1 mM EDTA, with protease inhibitors, pH 6.0 at final concentrations in the range 0.5–1 mM. DSS was used for spectra referencing.

Microtubule proteins were prepared from calf brains by repeated cycles of assembly– disassembly using a temperature-dependent procedure. Native tubulin heterodimers were purified following our published protocols and their viability was checked by non-denaturing electrophoresis, as described [49], [50]. The solution of αβ-tubulin dimer was dissolved in 50 mM MES buffer, 1 mM EGTA, 0.25 mM MgCl_2_, pH 6.7. The concentration of the stock solution was ∼30 µM. Two concentrated solutions of peptides derived from the C-terminal end of the β6-tubulin (Class III) monomer (^412^ESNMNDLVSEYQQYQDATAD^431^ and ^435^EMYEDDEEESESQGPK^450^) [17] were prepared in water to final concentrations of 7 mM and 10 mM, respectively.

Spectral assignment was done using sets of standard 2D and 3D experiments as reported [14]. For the backbone ^15^N-^1^H NOE measurement, experiments with and without proton saturation were acquired simultaneously in an interleaved manner and split during processing into separate spectra for analysis. A relaxation delay of 7.5 s was used. The NOE values were obtained from the ratio intensities of the resonances in both spectra.

The TBCC N-terminal domain interaction with αβ-tubulin was followed by comparing the ^15^N-HSQC spectrum of the free TBCC N-terminal domain, with that obtained after the addition of ∼100 µL of the TBCC N-terminal domain to the tubulin stock solution. Both the chemical shift and line width changes were analysed. To test the interaction with the β-tubulin peptides, the appropriate volumes of the concentrated solutions were added to the TBCC N-terminal domain sample to get approximately 1∶1 protein∶peptide stoichiometries. Changes of peak intensity and position were monitored. In all cases the pH was checked at the final points of the titrations.

All the experiments were recorded at 25°C on a Bruker AV 800 NMR spectrometer equipped with a cryoprobe. The spectra were processed with Bruker Topspin (Bruker, Germany) and spectral analysis was performed with Sparky3 [51]. Molmol [52] was used for molecular display.

### Structure calculation

The structure calculation of the TBCC N-terminal domain was performed with CYANA [53] using the automatic NOE assignment facility combined with lists of manually assigned NOEs. In total there were 1692 upper distance constraints, 870 of which were manually assigned. Backbone dihedral angle constraints were determined, for each residue except for the segments M25-R32, K46-Q48, P133-K135, and A78, E79, and L131, from chemical shift values using TALOS+ [54]. Initially 100 conformers were generated that were forced to satisfy experimental data using a standard automatic CYANA protocol [53]. The 20 conformers with the lowest final CYANA target function values were selected and subjected to 2,000 steps of energy minimization using the generalized Born continuum solvation model [55] implemented in AMBER9 [56] with a non-bonded cutoff of 10 Å. The AMBER energy was −7,000 kcal/mol with an electrostatic contribution term of −6,300 kcal/mol. Final structure quality was checked with PROCHECK-NMR [57] and the coordinates have been deposited in the PDB under the accession number 2l3l. Statistics of the calculation are summarized in Table 1.
